# Job requirements and physical demands (JRPD) questionnaire: cross-cultural adaptation and psychometric evaluation in Iranian Army personnel with chronic low back pain

**DOI:** 10.1186/s12891-021-04961-8

**Published:** 2022-01-05

**Authors:** Mehdi Ramezani, Ehsan Pourghayoomi, Ghorban Taghizadeh

**Affiliations:** 1 Department of Neuroscience, Faculty of Advanced Technologies in Medicine, Iran University of Medical Sciences, Tehran, Iran; 2 Department of Occupational Therapy, Rehabilitation Research Center, School of Rehabilitation Sciences, Iran University of Medical Sciences, Tehran, Iran

**Keywords:** Cross-cultural adaptation, Psychometric properties, Validity and reliability, Persian, Army personnel, Low back pain

## Abstract

**Background:**

Biomechanical risk factors have been identified as the main predisposing factor of chronic low back pain (CLBP), especially in Army personnel. The Job Requirements and Physical Demands (JRPD) questionnaire has been developed to assess the biomechanical exposures related to CLBP. Examining the biomechanical risk factors could prevent CLBP. This study aimed to translate and cross-culturally adapt the JRPD into Persian and assess its psychometric properties among Iranian male Army personnel with CLBP.

**Methods:**

In this cross-sectional study, the content validation of the JRPD was assessed after translating to Persian. The Persian JRPD was administered to 198 male Army personnel with CLBP, with an interval of 7 days, to assess test-retest reliability, including Cronbach’s α, intraclass correlation coefficients (ICC), standard error of measurement (SEM), and minimal detectable change at 95% confidence interval (MDC_95%_). Scores of the Persian JRPD were correlated with the scores of visual analog scale (VAS), Borg’s category-ratio (CR10) scale, general health questionnaire-28 (GHQ-28), and physical functioning (PF_1_ and PF_2_) subscale of the 12-item short-form health survey (SF-12) to assess convergent validity using Spearman correlation for a priori hypotheses.

**Results:**

The Persian JRPD had good content validity evidenced by the higher content validity index (> 0.70). The questionnaire had a significant positive negligible to weak correlation with the VAS (rho = 0.27; *p* < 0.001), Borg’s CR10 scale (rho = 0.19; *p* = 0.009), and the total score of GHQ-28 and its domains (rho ≤0.34; *p* < 0.05); and significant negative weak correlation with PF_2_ (rho = − 0.27; *p* < 0.001) and significant negative moderate correlation with PF_1_ (rho = − 0.35; *p* < 0.001), thus confirming the priori hypotheses (89%, 8/9). The internal consistency and ICC (*α* = 0.91; ICC = 0.80) were highly adequate, with SEM and MDC_95%_ of 7.91 and 21.3 respectively.

**Conclusions:**

The JRPD was successfully adapted into Persian and had adequate psychometric properties in terms of content and convergent validity, internal consistency, and test-retest reliability. The questionnaire is found useable to assess the CLBP-related biomechanical exposures in Iranian male Army personnel.

## Background

Low back pain (LBP) is the most common disorder among Army personnel, defined as pain localized below the costal margin and above the inferior gluteal folds, with or without leg pain [1–3]. LBP impacts troop readiness and leads to ambulatory care, work duty limitation, lost days, and disability in the Armed forces [4–7]. The LBP prevalence is rising, and the LBP-related costs have increased substantially over the past decades [8]. 4 to 19*%* of patients with LBP feel the pain for 3 months or longer, called chronic LBP (CLBP) [8, 9]. CLBP is a multifactorial disorder, and previous studies have confirmed the roles of individual factors, health behaviors, work organization, psychosocial, and biomechanical (occupational) risk factors in the back pain onset and its exacerbation [4, 5, 10]. Overall, the LBP-related risk factors in Army personnel are categorized as individual, psychosocial, and occupational risk factors [3]. Despite the critical role of the individual and psychosocial factors in developing LBP, Army personnel are at high risk of developing LBP due to job demands [3]. Army occupations involve heavy physical activities, causing a higher-than-average chance of disability [3, 11]. Army-related activities, such as repetitive heavy lifting, frequent twisting/bending, forceful pushing and pulling, and awkward body postures are associated with the CLBP [5, 7, 11, 12]. Examining the occupational risk factors could prevent CLBP [5, 13]. Also, the primary prevention programs would enhance the performance and quality of life in the Army personnel [7, 14].

Self-report questionnaires as an examining method to measure biomechanical exposures have reduced the resources of time and costs [5]. Various self-report questionnaires are currently available for the evaluation of LBP, such as Job Requirements and Physical Demands (JRPD) [5, 7, 15], Roland–Morris disability questionnaire and its variants [16, 17], Oswestry disability index and its several versions [17, 18], the Quebec back pain disability scale [19], the Waddell disability index [20], the low back outcome score [21], and many other measures (see further information in Longo et al. [17]). All these questionnaires, except JRPD, estimate the degree of patients’ disabilities in physical and mental functions during daily living, productivity, and work quality. However, the JRPD questionnaire is an exclusive scale to measure the LBP-related biomechanical exposures.

Based on our best knowledge, the JRPD questionnaire has not already been translated or cross-culturally adapted into other languages. Concerning the high prevalence of LBP in Iranian military staff (96% had different degrees of LBP [22]), in the current study, we aimed to translate the original JRPD questionnaire to the Persian language, perform the cross-culturally adaptation, and evaluate the validity and reliability of the questionnaire in a sample of Iranian male Army personnel who suffered from CLBP.

## Methods

### Study design and participants

This cross-sectional study was performed using a convenient sampling method [23]. Of selected Iranian Army centers in Tehran province, 198 male patients with CLBP participated in the current study from February 2013 to August 2018. The LBP with or without leg pain of at least 3 months duration, age 18 years or older, and fluency in Persian language were the inclusion criteria of the study. Exclusion criteria were previous or scheduled lumbosacral spine surgery, LBP due to trauma, obvious structural deformity, psychological conditions, and suspected or confirmed specific serious conditions of the spine, such as inflammatory or infective diseases, fracture, malignancy, osteoporosis, cauda equina syndrome, and nerve compression due to herniated disc or spinal stenosis. Female gender, participants who answered the questionnaires incompletely in the test or retest phase, and individuals with a visual analog scale (VAS) score of ≤4 mm, as without CLBP, were excluded from the study. Participants were interviewed and screened for inclusion/exclusion criteria; the participants’ available medical records were also reviewed to obtain data.

### Translation procedure

**Fig. 1:**
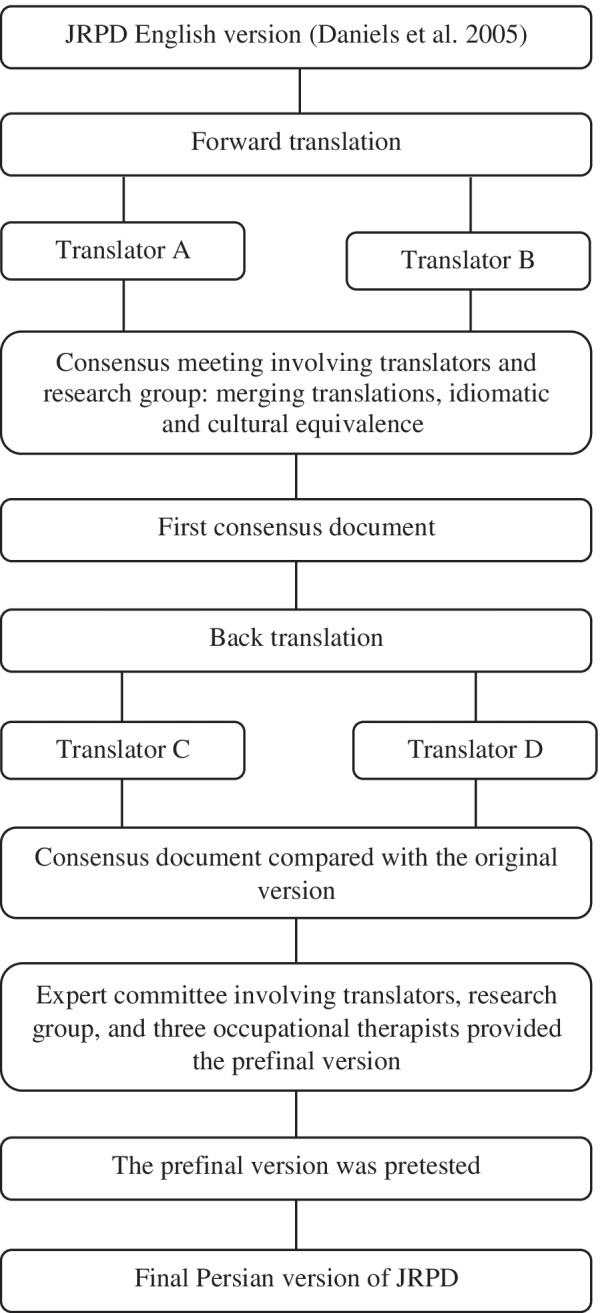
A flow chart illustrating the procedure of translating the English-version of Job Requirements and Physical Demands (JRPD) questionnaire to the Persian language

Before the study, we obtained permission to translate the self-report JRPD into the Persian language from the authors who converted it to a self-report. The cross-cultural adaptation process was performed according to the guidelines proposed by Beaton et al. [24] to provide a linguistically and culturally equivalence between the original and translated version (Fig. 1). The first step of the cross-cultural adaptation was the forward translation, in which the questionnaire was translated from the English language to Persian. Two bilingual Persian-native expert translators produced two independent translations. Item content, response options, and instructions were all translated. One of the translators was aware of the concepts being examined, whereas the other translator was not informed of the concepts being quantified and had no clinical background. They provided written reports, included additional comments highlighting challenging phrases or uncertainties and their rationale for choices. In the second step, translators and research administrators synthesized the translations and formulated an initial version of Persian JRPD by comparing the translated texts and solving all the discrepancies.

In the third step (backward translation), two bilingual English-native expert translators translated the initial version of Persian JRPD back into English. They were blinded to the original version, not informed of the concepts explored, and had no clinical background. In the fourth step, the expert committee consisting of all translators, research administrators, and three occupational therapists reviewed all the versions, reached a consensus about all the discrepancies, and eventually provided the prefinal version of Persian JRPD. In the final step, the prefinal version was pilot tested on 70 Army personnel with CLBP (see further details at [7]) to determine the qualitative face validity [25]. The qualitative face validity involves a face-to-face interview to find ambiguous, confusing, irrelevant, unclear, or redundant items [25]. Participants reported no difficulties in the completion of the prefinal Persian version of JRPD during the pilot study (good face validity) [7]. Ultimately, the final version of Persian JRPD was provided for psychometric assessments.

### Instruments

A self-administered questionnaire was used to gather participants’ demographic data. Furthermore, the self-report scales, including the Persian JRPD, pain VAS, Borg’s category-ratio (CR10) scale, general health questionnaire (GHQ-28), and two physical functioning (PF) items (PF_1_ and PF_2_) of the 12-item short-form health survey (SF-12), were used to collect data. The questionnaires were completed by the participants, on paper, in the presence of the researcher.

Job requirements and physical demands (JRPD) questionnaire: The JRPD is a valid measure of back pain-related biomechanical exposures, consisting of 38 items that examine both types of exposure and duration of biomechanical exposures [5, 7, 15, 26, 27]. Each item is rated on a 5-point Likert scale: 1 (never), 1 (≤ 5 h/week), 2 (≤ 2 h/day), 3 (2 to 4 h/day), and 4 (≥ 4 h/day) [5, 7, 15, 27]. The total score (range: 38–152) is obtained by summing the scores of all the 38 items, with higher scores indicating higher levels of biomechanical exposure and a greater likelihood of a subject suffering from LBP within the past 12 months [5, 26].

Visual analog scale (VAS): This scale is a valid and reliable measure of pain intensity that has been widely used in various adult study populations. It is a 100-mm horizontal line anchored on the left with the phrase “No pain” and on the right with the phrase “pain as bad as it could or worst imaginable pain” [28]. Participants were asked to mark a point on the horizontal line that best represents their level of pain intensity. A ruler was used to measure the distance between 0-mm and the patient’s marked point to determine the patient’s score [28]. The cut-points for VAS have been recommended: no pain (0–4 mm), mild pain (5–44 mm), moderate pain (45–74 mm), and severe pain (75–100 mm) [29].

Borg’s category-ratio (CR10) scale: This scale is a valid tool for rating the levels of physical or muscular fatigue and whole body exertion due to work [26]. It consists of 10 numerical lists, with each number representing the participant’s level of exertion during activity. The numbers on the scale are defined as: 0 (no exertion at all), 0.5 (very, very slight (just noticeable)), 1 (very slight), 2 (slight), 3 (moderate), 4 (somewhat severe), 5 (severe), 6 and 7 (very severe), 8 and 9 (very, very severe (almost maximal)), and 10 (maximal exertion) [30]. A higher score indicates a higher load of both cardiovascular and muscular work [30]. Participants were asked to choose a number that best reflects their whole body exertion due to work [26].

General health questionnaire (GHQ-28): This is a valid measure developed to identify minor psychiatric and psychological disorders. The Persian version of the GHQ-28 was used in the current study [31]. It is a 28-item questionnaire comprising of domains of somatic symptoms (items 1–7), anxiety/insomnia (items 8–14), social dysfunction (items 15–21), and severe depression (items 22–28). Each item is rated on a 4-point Likert scale. A higher score implies a higher unfavorable psychological status [31, 32].

Short-form health survey (SF-12): SF-12 is a valid and reliable measure of the impact of health on an individual’s everyday life [33]. The questionnaire assesses overall physical and mental health outcomes, and it is commonly used in various medical studies on patients with a variety of chronic conditions [33]. In the current study, the two PF items of the Persian version of SF-12 (PF_1_: limitations in moderate physical activities; and PF_2_: limitations in climbing several flights of stairs) were used [33]. Also, instead of the mental health items of the SF-12, the GHQ was used to assess mental health thoroughly.

### Assessment of psychometric properties

The present study examined the psychometric properties of Persian JRPD, including the floor/ceiling effects, content and convergent validity, internal consistency, and test-retest reliability.

Floor/ceiling effects: Acceptability of the Persian JRPD was assessed by determining floor/ceiling effects. The floor/ceiling effects were considered to occur if more than 10% of the participants achieved the minimum or maximum possible score on the scale [23].

Content validity: Following translation, content experts were invited to participate in the study for assessing Persian JRPD content validation. There is no clear idea of the ideal number of content experts needed in a validation study [34]. However, six experts are usually considered an adequate number [35, 36]. In the current study, three occupational therapists and four physiotherapists, with our participants’ same language and culture, reviewed all items of the questionnaire for relevancy, simplicity, clarity, and the necessity of each item [37]. After receiving a questionnaire that included questions related to content validity, they had 7 days to respond to the questions [34]. For assessing the quantitative content validity of each item of the questionnaire, the content validity index (CVI) and content validity ratio (CVR) were calculated based on the Lawsheis model [37]. Content experts were asked to declare their level of agreement for relevancy, simplicity, clarity, and the necessity of each item as to which items should be included in the final Persian JRPD. Each item of the questionnaire was rated using a 4-point Likert scale [38]. Acceptable values for CVI and CVR were considered higher than 0.70 and 0.59, respectively [37, 39].

Convergent validity: Convergent validity of the Persian JRPD was explored by correlation, using Spearman correlation coefficient and comparing the Persian JRPD with pain VAS, Borg’s CR10 scale, GHQ-28, and two PF_1_ and PF_2_ items of the SF-12. We hypothesized that the Persian JRPD would have positively negligible to weak correlations with the Borg’s CR10 scale [5], the pain VAS [5], and the total score of the GHQ-28 and its domains [40, 41]. Also, we hypothesized that the Persian JRPD would have negatively negligible to weak correlations with two PF_1_ and PF_2_ items of the SF-12 [5]. Nine correlations were analyzed, and the convergent validity was considered adequate if > 75% (7 out of 9) of the predefined hypotheses were verified. Correlation values of 0.00 to 0.19, 0.20 to 0.34, 0.35 to 0.50, and > 0.50 were interpreted as negligible, weak, moderate, and strong correlation, respectively [42].

Internal consistency: Internal consistency was estimated through Cronbach’s alpha (α). Alpha values ≥0.90, 0.90 > α ≥ 0.80, 0.80 > α ≥ 0.70, 0.70 > α ≥ 0.60, 0.60 > α ≥ 0.50, and alpha < 0.50 were interpreted as excellent, good, acceptable, questionable, poor, and unacceptable inter-item reliability, respectively [43].

Test-retest reliability: Intraclass correlation coefficient (ICC) and standard error of measurement (SEM) were used to calculate the relative and absolute reliability, respectively. Since the Persian JRPD is a self-report questionnaire, the effect of the observer/rater in answering the items is minimum. Accordingly, the test-retest relative reliability of the questionnaire was estimated based on a mean-rating (k = 3), absolute-agreement, two-way mixed-effects model (ICC _3, 1_), with a 95% confidence interval [44]. Values less than 0.50, 0.50 to 0.75, 0.75 to 0.90, and greater than 0.90 are indicative of poor, moderate, good, and excellent reliability, respectively [44]. The SEM was calculated using the formula of *SD*_*pooled*_ *× √1-ICC*. The SD _pooled_ is the standard deviation of the total score of the questionnaire for all participants [45]. An SEM value of less than half of SD _pooled_ is considered acceptable [46]. Also, minimal detectable change at 95% confidence interval (MDC_95%_) of the questionnaire was calculated using the formula of *±1.96 × √2× SEM* [47]. The value of 1.96 is a *z* score associated, with a 95% confidence interval [45, 47]. MDC_95%_ determines the minimal change which falls outside the measurement error in the score of a questionnaire [48, 49]. A questionnaire with a smaller MDC_95%_ is sufficiently sensitive [47]. By considering participants were not aware of the completion of the questionnaire again, they responded to the questions with a seven-day interval [50]. Participants were asked to complete the questionnaire without the rater’s assistance.

### Statistical analysis

Descriptive statistics of means, standard deviation, frequency, and percentages were used to summarize quantitative and qualitative variables. The normal distribution of data was tested using the Shapiro Wilk test [51]. Since the result of the normality test for the total score of the Persian JRPD was skewed, Spearman’s correlation coefficient was used to measure the degree of correlation between the Persian JRPD total scores and other variables. All analyses were performed using a statistical package for the social sciences (SPSS 21.0, Chicago, IL) with the statistical significance level of *p* < 0.05 and 95% confidence interval.

## Results

One hundred and ninety-eight male Army personnel with a mean (SD) age of 32 (10.4) years participated in the present study. The demographic and clinical characteristics of participants are shown in Table 1.

**Table 1:** Demographic characteristics of the study population (*N* = 198, female = 0)

Characteristics	Mean (SD)
Age (year)	32 (10.40)
Height (cm)	175 (6.80)
Weight (kg)	74 (1.10)
Pain intensity (VAS)	36 (25.30)
	Frequency (%)
Mild pain (5–44 mm)	128 (64.60)
Moderate pain (45–74 mm)	49 (24.80)
Severe pain (75–100 mm)	21 (10.60)
Educational status	
Academic	151 (76.30)
Non-academic	47 (23.70)
Marital status	
Single	82 (41.40)
Married	116 (58.60)
Employment status	
Employed	128 (64.60)
Unemployed	70 (35.40)
Service status	
Military	139 (70.20)
Non-military	59 (29.80)
Military ranks	
Colonel	6 (3.00)
Major	10 (5.10)
Captain	15 (7.60)
Lieutenant	126 (63.70)
Master sergeant	11 (5.50)
Sergeant	5 (2.50)
Soldier	25 (12.60)

*Abbreviations*: *SD* standard deviation, *Min* minimum, *Max* maximum, *VAS* visual analog scale

Eighty-four out of the 198 (42.4*%*) participants had a minimum (floored) total score, and none had a maximum (ceiling) total score. The CVI for items of the Persian JRPD ranged from 0.76 to 1.00. All the 38 items had CVI higher than 0.70, which implies a good content validity for these items. Twelve out of 38 items had a CVI of 1, indicating a complete agreement among the content experts. The CVR for items of the Persian JRPD ranged from 0.60 to 1.00, and 14 out of 38 items had a CVR of 1, indicating a complete agreement among the content experts. Content validation outcomes of the Persian JRPD questionnaire are presented in Table 2.

**Table 2:** Content validation outcomes of the Persian JRPD questionnaire (*N* = 7)

Item	Description	CVI	CVR
1	I work with my hands at or above chest level	0.93	1.00
2	To get to or do my work, I must lay on my back or side and work with my arm up	0.93	0.90
3	I must hold or carry materials (or large stacks of files) during the course of my work	1.00	1.00
4	I force or yank components of work objects in order to complete a task	0.90	0.90
5	I reach / hold my arms in front of or behind my body (e.g. Using keyboard, filing, handling parts, perform inspection tasks, pushing/ pulling carts, etc.)	1.00	0.90
6	My neck is tipped forward or backward when I work	0.96	1.00
7	I cradle a phone or other device between my neck and shoulder	1.00	0.70
8	My wrists are bent (up, down, to the thumb, or little finger side) while I work	0.93	1.00
9	I apple pressure or hold an item /material /tool (e.g., screwdriver, spray gun, mouse, etc. in my hand for longer than 10 s at a time)	0.96	0.90
10	My work requires me to use my hands in a way that is similar to wringing out clothes	1.00	0.90
11	I perform a series of repetitive tasks/ movement during the normal course of my work (e.g. using keyboard, tightening fastener, cutting meat, etc.)	0.93	1.00
12	The work surface (e.g., desk, bench, etc.) or tool(s) that I use presses into my palm(s),wrist(s), or against the sides of my fingers leaving red marks on or beneath the skin	0.93	1.00
13	I use my hand/ palm like a hammer to do aspects of my work	0.96	1.00
14	My hands and fingers are cold when I work	0.90	0.80
15	I work at a fast pace to keep up with the machine production quota or performance incentive	0.83	0.80
16	The tool(s) that I use vibrates and/ or jerks my hand(s)/arm(s)	1.00	1.00
17	My work requires that I repeatedly throw or toss items	0.96	0.90
18	My work requires me to twist my forearms, such as turning a screwdriver	1.00	1.00
19	I wear gloves that are bulky, or reduce my ability to grip	0.96	0.70
20	I squeeze or pinch work objects with a force similar to that which is required to open a lid on a new jar	0.90	0.80
21	I grip work objects or tools as if I am griping tightly onto a pencil	0.86	0.80
22	When I lift, move components, or do other aspects of my work, my hands are lower than my knees	0.83	1.00
23	I lean forward continually when I work (e.g., when sitting, when standing, when pushing carts, etc.)	0.93	0.90
24	The personal protective equipment or clothing that I wear limits or restricts my movement	1.00	0.80
25	I repeatedly bend my back (e.g., forward, backward, to the side, or twist) in the course of my work	1.00	1.00
26	When I lift, my body is twisted and/ or I lift quickly	0.96	0.80
27	I can feel vibration through the surface that I stand on, or through my seat	0.90	0.90
28	I lift and/ or carry items with my hand	1.00	0.90
29	I lift or handle bulky items	1.00	1.00
30	I lift materials that weigh more than 25 pounds	0.96	0.90
31	My work requires that I kneel or squat	1.00	1.00
32	I must constantly move or apply pressure with one or both feet (e.g. using foot pedals, driving, etc.)	0.96	0.90
33	When I’m sitting, I cannot rest both feet flat on the floor	0.80	1.00
34	I stand on hard surface	1.00	0.90
35	I can see glare on my computer screen or work surface	0.76	0.60
36	It is difficult to hear a person on the phone or to concentrate because of other activity, voices, or noise in/ near my work area	0.83	0.60
37	I must look at the monitor screen constantly so that I do not miss important information (e.g. radar scope)	0.93	0.60
38	It is difficult to see what I am working with (monitor, paper, parts, etc.)	0.76	0.60

*Abbreviations*: *JRPD* Job requirements and physical demands, *CVI* content validity index, *CVR* content validity ratio

As hypothesized, the Spearman correlation analyses indicated significant positive negligible to weak correlations between the Persian JRPD and VAS, Borg’s CR10 scale, and the total score of GHQ-28 and its domains. As hypothesized, a significant negatively weak correlation between the Persian JRPD and PF_2_ was estimated; however, the Persian JRPD and PF_1_ were correlated moderately (Table 3).

**Table 3:** Correlation of the Persian JRPD total score with other scales (*N* = 198)

Variable	Spearman	*p-value
VAS	0.27	**< 0.001**
SF-12		
PF_1_	- 0.35	**< 0.001**
PF_2_	- 0.27	**< 0.001**
GHQ-28 (*N* = 151)		
Total score	0.31	**< 0.001**
Somatic symptoms (1–7 items total score)	0.22	**0.007**
Anxiety/insomnia (8–14 items total score)	0.33	**< 0.001**
Social dysfunction (15–21 items total score)	0.28	**< 0.001**
Severe depression (22–28 items total score)	0.21	**0.009**
Borg’s CR10 scale	0.19	**0.006**

*Abbreviations*: *JRPD* job requirements and physical demands, *VAS* visual analog scale, *SF-12* standard short form health survey, *PF*_1_ physical functioning-1 (limitations in moderate physical activities), *PF*_2_ physical functioning-2 (limitations in climbing several flights of stairs), *GHQ* general health questionnaire. Bolded values present significant difference (*p < 0.01)

The mean (SD) of the Persian JRPD total score in the pretest and posttest were 56 (17.0) and 53 (18.1), respectively. The internal consistency as measured by Cronbach’s alpha was 0.91. ICC _(3, 1)_ value for Persian JRPD total score was found to be 0.80. The SEM and MDC_95%_ for the Persian JRPD total score were 7.91 and 21.3, respectively (Table 4).

**Table 4:** Reliability outcomes of the Persian JRPD questionnaire at a 95% confidence interval (*N* = 198)

Items	Mean (SD)	*Sig	Cronbach’s Alpha Coefficient	ICC _3,1_ (upper band–lower band)	SEM	MDC
38	56 (17.02)	**< 0.001**	0.91	0.80 (0.73–0.84)	7.91	21.32

*Abbreviations*: *JRPD* job requirements and physical demands, *SD* standard deviation, *Sig* significance, *ICC* intraclass correlation coefficient, *SEM* standard error measurement, *MDC* minimal detectable change. Bolded value presents significant difference (*p < 0.05)

## Discussion

This is the first study to report the cross-cultural adaptation of the JRPD questionnaire into Persian and its validation. The results of the study suggest that the Persian version of the JRPD is a valid and reliable questionnaire when tested among a sample of Iranian male Army personnel with CLBP.

Floor effects were observed (42.4%) for the total score of the Persian JRPD, suggesting a measuring limitation of the questionnaire. The flooring effect may reduce the sensitivity of scale and distorts the ability of the questionnaire to detect any real change after interventions. Although the flooring effect can be an indicator of a weak content validity or poor reliability [52], our findings showed an acceptable content validity and reliability for the Persian JRPD (more discussion below). Therefore, the observed floor effects could not be due to poor validity or reliability. Usually, the floor effect is because of inherent weaknesses in the measurement/scoring system [53]. It appears that the obtained flooring effect stemmed from the existence of various items in the Persian JRPD questionnaire. Observing many items of the JRPD during a certain work is impossible. In other words, just 1 out of 38 items of the questionnaire, as the physical requirements for work, would be enough to suffer back pain. Consistent with this statement, it has been shown in previous studies that three activities (3 selected items of the JRPD) in mothers of children with cerebral palsy and five activities (5 items selected from JRPD) in the military were associated with CLBP [7, 15]. Hence, we suggest developing a short-form of the Persian JRPD in different jobs.

All the 38 items of the Persian JRPD questionnaire had an acceptable CVI and CVR values, suggesting a good content validity. Consequently, the number of items in the Persian JRPD remained unchanged. Therefore, all items of the questionnaire were relevant to the assessment of biomechanical exposures, which would lead to CLBP in Iranian Army personnel. Content validation is critical in developing a questionnaire [36, 54], and if the questionnaire lacks content validity, it is impossible to establish reliability [36, 55]. The content validity also provides preliminary evidence on the construct validity of the instrument [36]. Based on this piece of literature, the content validity finding for Persian JRPD shows that the Persian JRPD is a valid questionnaire.

Convergent validity refers to the correlation between measures [56]. For estimating the convergent validity (despite statistical significance), we have found a negligible correlation of the Persian JRPD with Borg’s scale. The Persian JRPD had a weak correlation with the VAS, PF_2_, and GHQ-28 total score and its domains. We have also found a moderate correlation between the Persian JRPD and PF_1_. Consequently, 8 out of 9 hypothesized correlations were accepted as negligible to weak correlations. In line with our results, Daniels and colleagues [5] have reported a negligible correlation of the original JRPD with pain intensity and physical/mental dysfunctions tested by SF-12. When two scales have a strong correlation, it implies that two measures capture equivalent information (strong convergent validity) [56]. Hence, the outcomes of the current study confirmed that the Persian JRPD questionnaire would not strongly capture the same information as the selected measures. It has been concluded that the Persian JRPD questionnaire, as a measure of CLBP-related biomechanical exposures, cannot be used instead of VAS, SF-12, GHQ-28, or Borg CR10 scale. Thus, it would be useful for future studies to examine the construct validity of the JRPD with other low back outcomes related to biomechanical exposures.

Our findings showed that the Persian JRPD had an excellent internal consistency, good relative reliability, and acceptable absolute reliability, indicating that the questionnaire is reliable for assessing CLBP-related biomechanical exposures among Iranian Army personnel. In line with our study, Daniels and colleagues [5] reported a high internal consistency (ICC = 0.95) for the English version of the JRPD. However, the authors did not examine the relative and absolute test-retest reliability of the JRPD. MDC_95%_ refers to the minimal amount of change outside of error in the total score of the questionnaire to determine whether a patient’s clinical outcome is getting better or getting worse [49, 57]. Our findings indicated the MDC_95%_ of 21.3 for the Persian JRPD. Therefore, researchers and clinicians should note that changes in the total score of Persian JRPD should exceed this amount to indicate an actual change in the health status. In other words, the test-retest difference less than this amount can be considered as a measurement error and should be ignored [58].

According to the literature, this is the first cross-cultural adaptation study of the JRPD questionnaire. Hence, we have not compared our results with versions of other languages. Furthermore, the present study has some limitations, which should be taken into considerations when interpreting the results. First, all data were collected by self-reported measures, thus, might be subject to recall bias. Second, we did not conduct factor analysis to explore the dimensionality of the Persian JRPD; this was due to the inadequate sample size, which is not usually recommended for factor analysis [59]. Lastly, external responsiveness, such as using an anchor-based method, was not performed in this study to determine whether changes in outcome scores are clinically relevant. Therefore, future studies should endeavor to explore the factor structure of the Persian JRPD for a possible shorter version and establish the minimally important change of the questionnaire. Additionally, future studies should examine the construct validity of Persian JRPD as most of the correlation coefficients obtained in this study, even though statistically significant, were generally weak or negligible. The authors also suggest conducting similar studies on different occupations and populations.

## Conclusion

The JRPD was successfully adapted into Persian and had adequate psychometric properties in terms of content and convergent validity, internal consistency, and test-retest reliability. The questionnaire can be used to assess the CLBP-related biomechanical exposures in Iranian Army personnel.

## Data Availability

The datasets used and/or analysed during the current study are available from the corresponding author on reasonable request.

## Abbreviations

LBP: Low Back Pain
CLBP: Chronic Low Back Pain
JRPD: Job Requirements and Physical Demands
MDC: Minimal Detectable Change
CVI: Content Validity Index
CVR: Content Validity Ratio
VAS: Visual Analog Scale
Borg’s CR10: Borg’s Category-Ratio scale
GHQ-28: General Health Questionnaire
PF: Physical Functioning
SF-12: 12-item Short Form survey
ICC: Intraclass Correlation Coefficient
SEM: Standard Error of Measurement
